# Supplementary figures and images for: Disruption of the Pseudomonas aeruginosa Tat system perturbs PQS-dependent quorum sensing and biofilm maturation through lack of the Rieske cytochrome bc1 sub-unit
Source: PLoS Pathog. 2021 Aug 30;17(8):e1009425. doi: 10.1371/journal.ppat.1009425 (PMC8432897; doi:10.1371/journal.ppat.1009425)

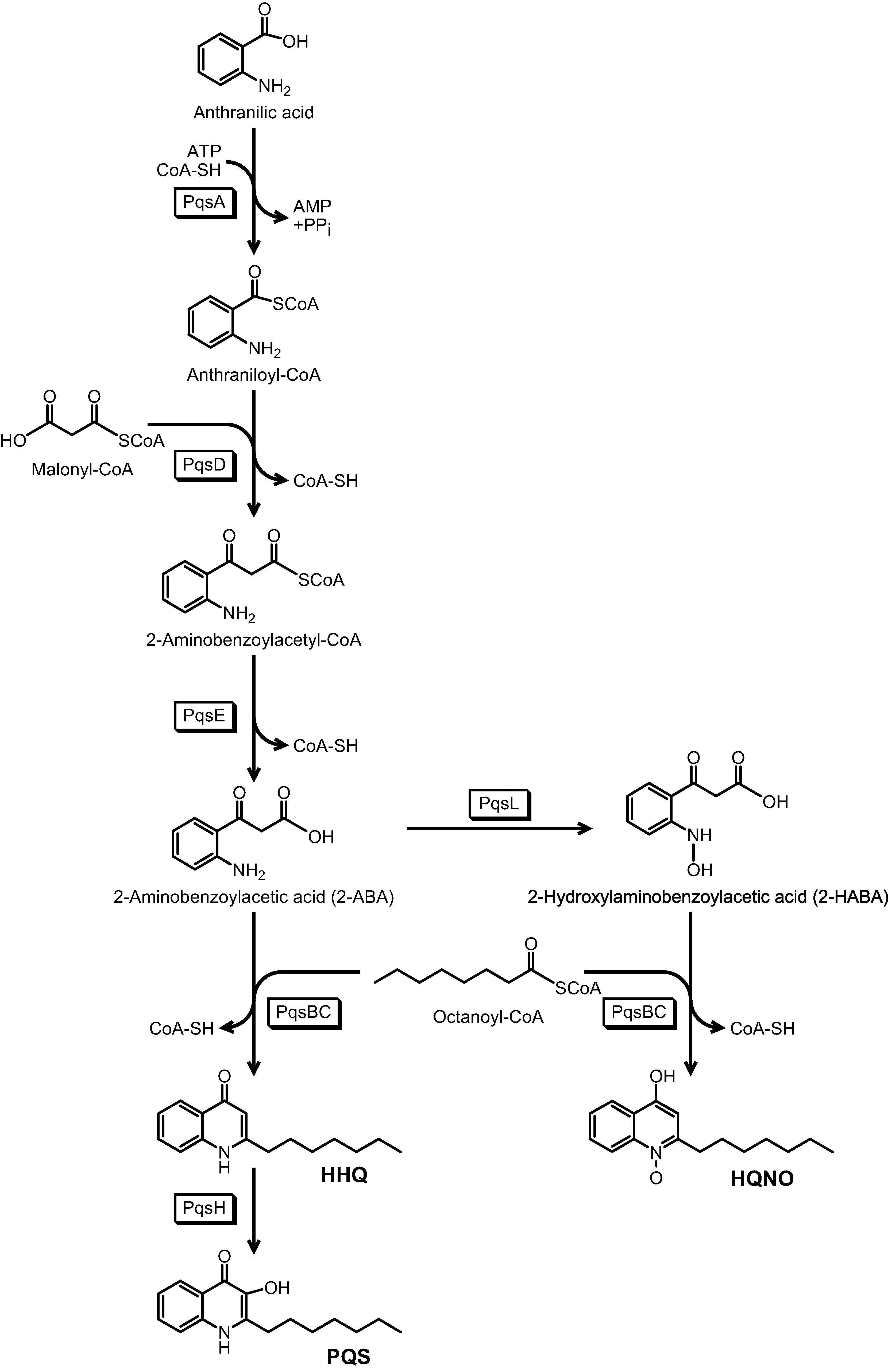

Supplement: S1 Fig — PqsA catalyses the formation of anthraniloyl-CoA that is condensed with malonyl-CoA by PqsD to form 2-aminobenzoylacetyl-CoA (2-ABA-CoA). The latter is converted to 2-aminobenzoylacetate (2-ABA) via the thioesterase functionality of PqsE. The PqsBC heterodimer condenses 2-ABA with octanoyl-CoA to generate HHQ. PQS is formed through the oxidation of HHQ by PqsH. For AQ N-oxides such as 2-heptyl-4-hydroxyquinoline N-oxide (HQNO), 2-ABA is oxidized to 2-HABA by the alternative mono-oxygenase PqsL and then condensed with octanoyl-CoA by PqsBC to form HQNO. PqsBC can accept acyl-CoAs of different acyl chain lengths to generate diverse AQs and AQ N-oxides. (TIF) [file ppat.1009425.s001.tif]

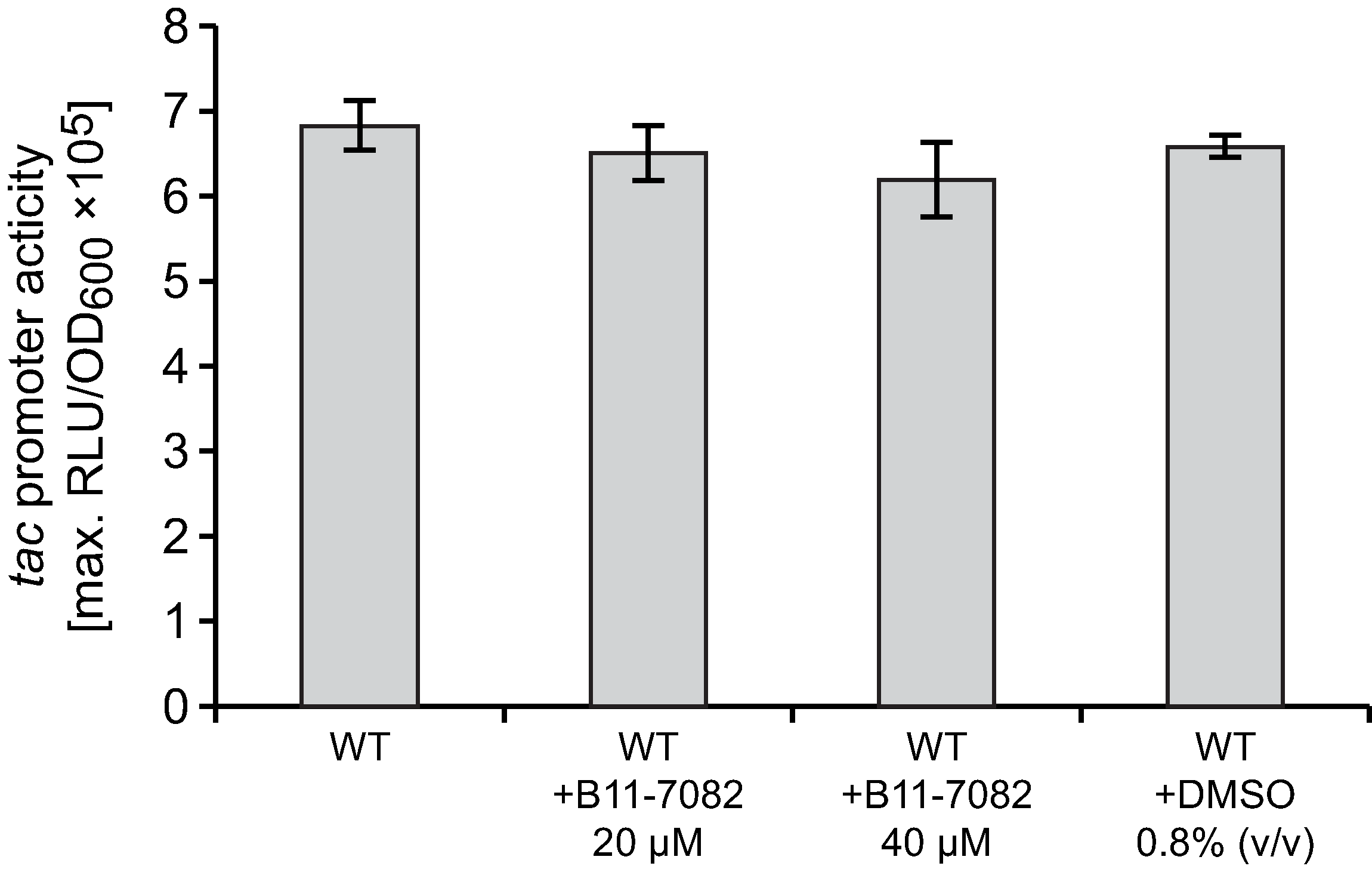

Supplement: S2 Fig — The co-solvent DMSO, had no effect at 0.4 or 0.8% on the lux reporter fusion Data are presented as maximal light output as a function of growth (RLU/OD600). Experiments were repeated in triplicate at least twice. (TIF) [file ppat.1009425.s002.tif]

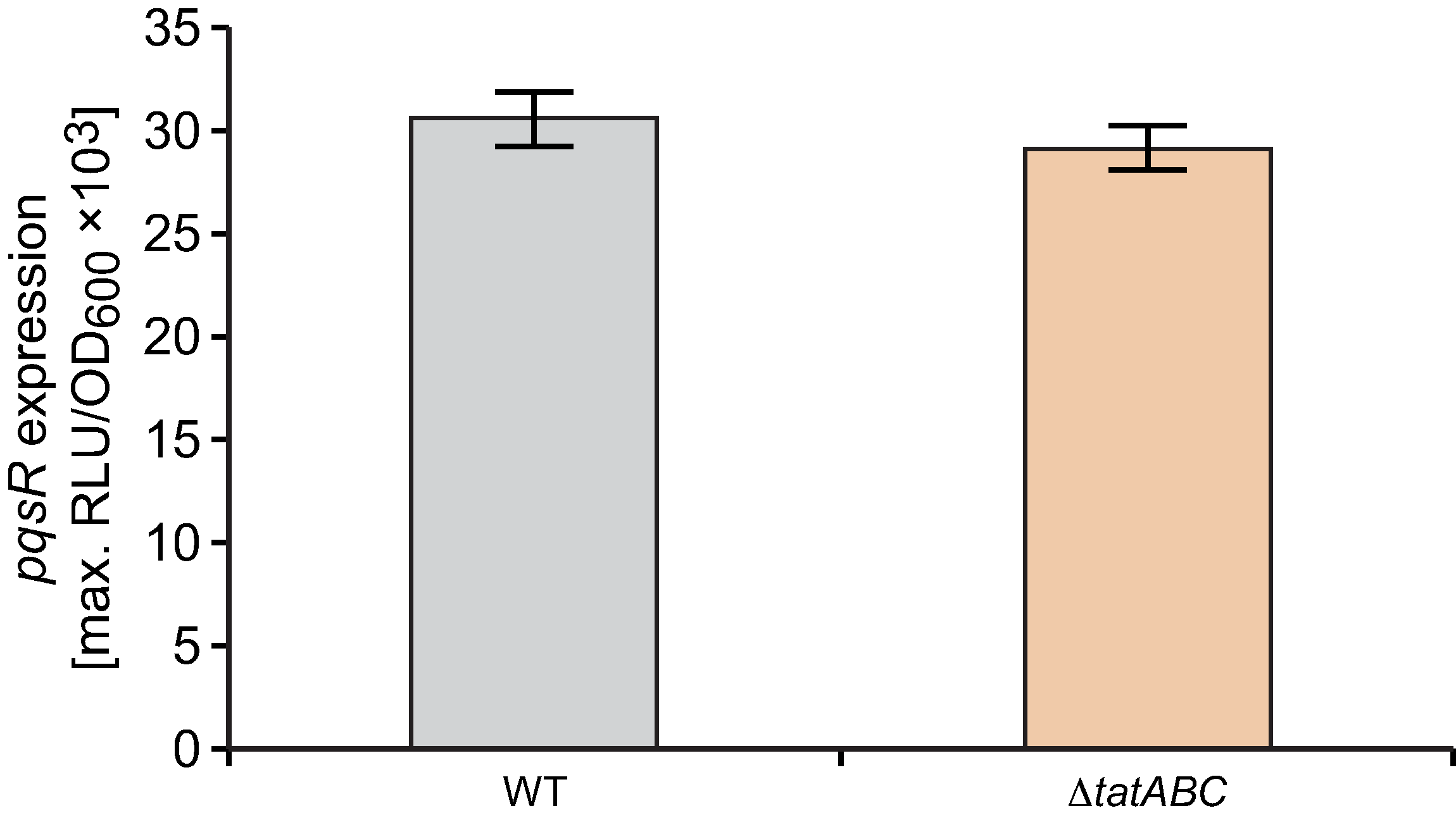

Supplement: S3 Fig — The data show that there are no differences in the expression of a chromosomal CTX::pqsR’-luxCDABE fusion in the P. aeruginosa wild type compared with the ΔtatABC mutant Data are presented as maximal light output as a function of growth (RLU/OD600). Experiments were repeated in triplicate at least twice. (TIF) [file ppat.1009425.s003.tif]

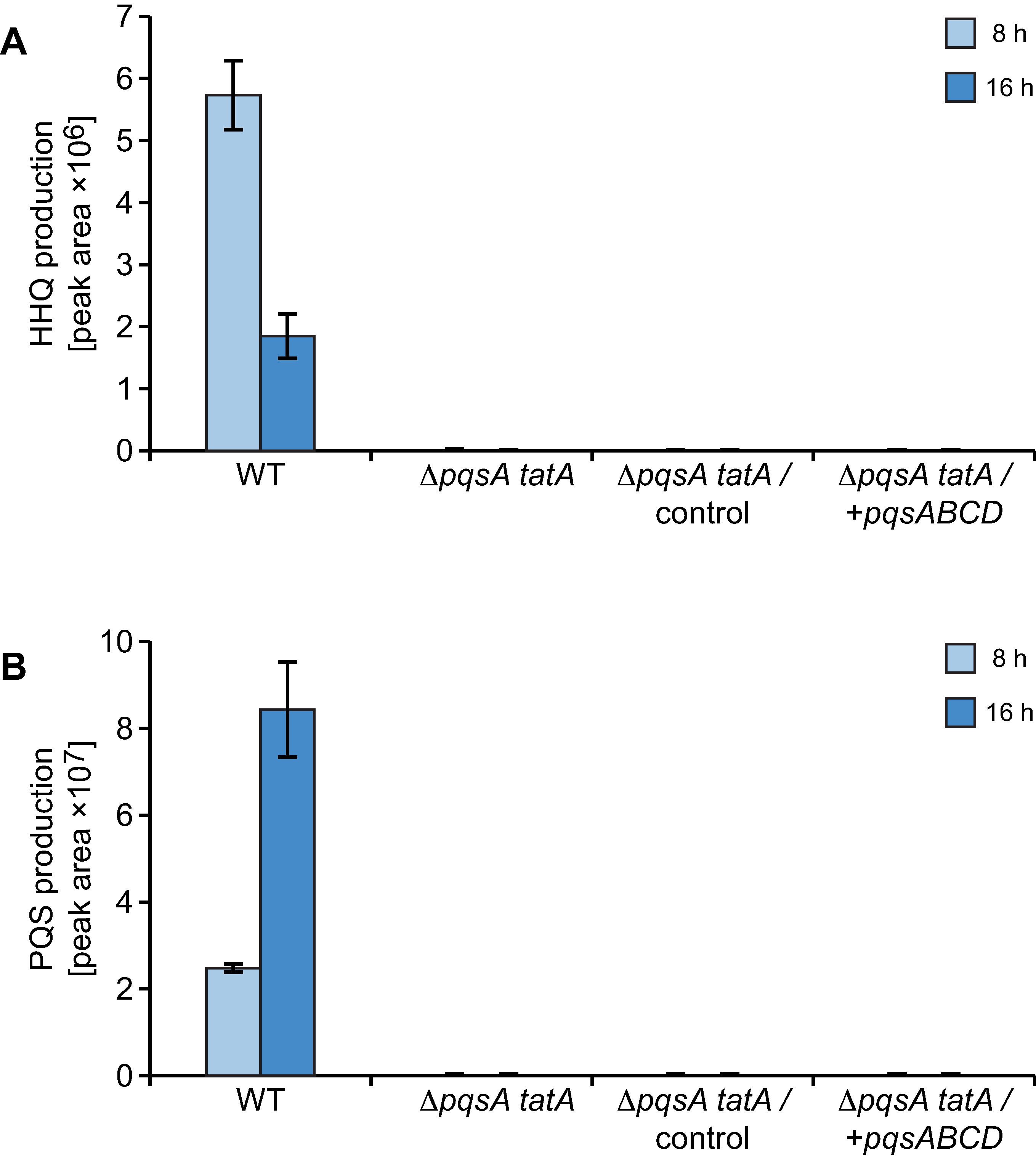

Supplement: S4 Fig — Semi-quantitative analysis by LC-MS/MS of PQS (A) and HHQ (B) extracted with methanol from whole cell cultures of P. aeruginosa wild type and the tatA ΔpqsA mutant without (control) or with (+pqsABCD) the pqsABCD biosynthetic genes provided via pBBR1MCS-5::pqsABCD and harvested at 8 h and 16 h respectively. Experiments were repeated in triplicate. (TIF) [file ppat.1009425.s004.tif]

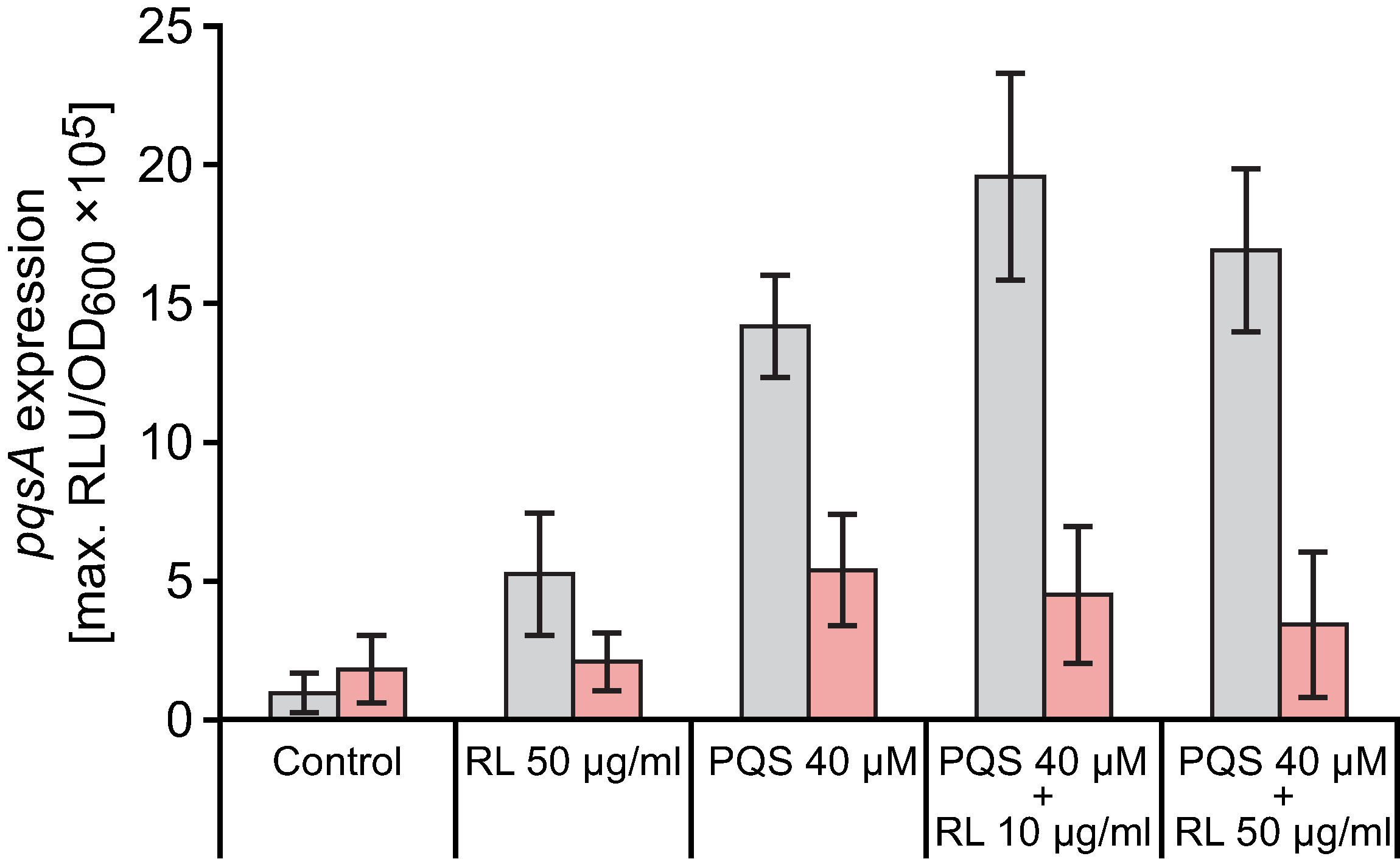

Supplement: S5 Fig — Maximal light output as a function of growth (RLU/OD600) is presented. Experiments were repeated in triplicate at least twice. (TIF) [file ppat.1009425.s005.tif]

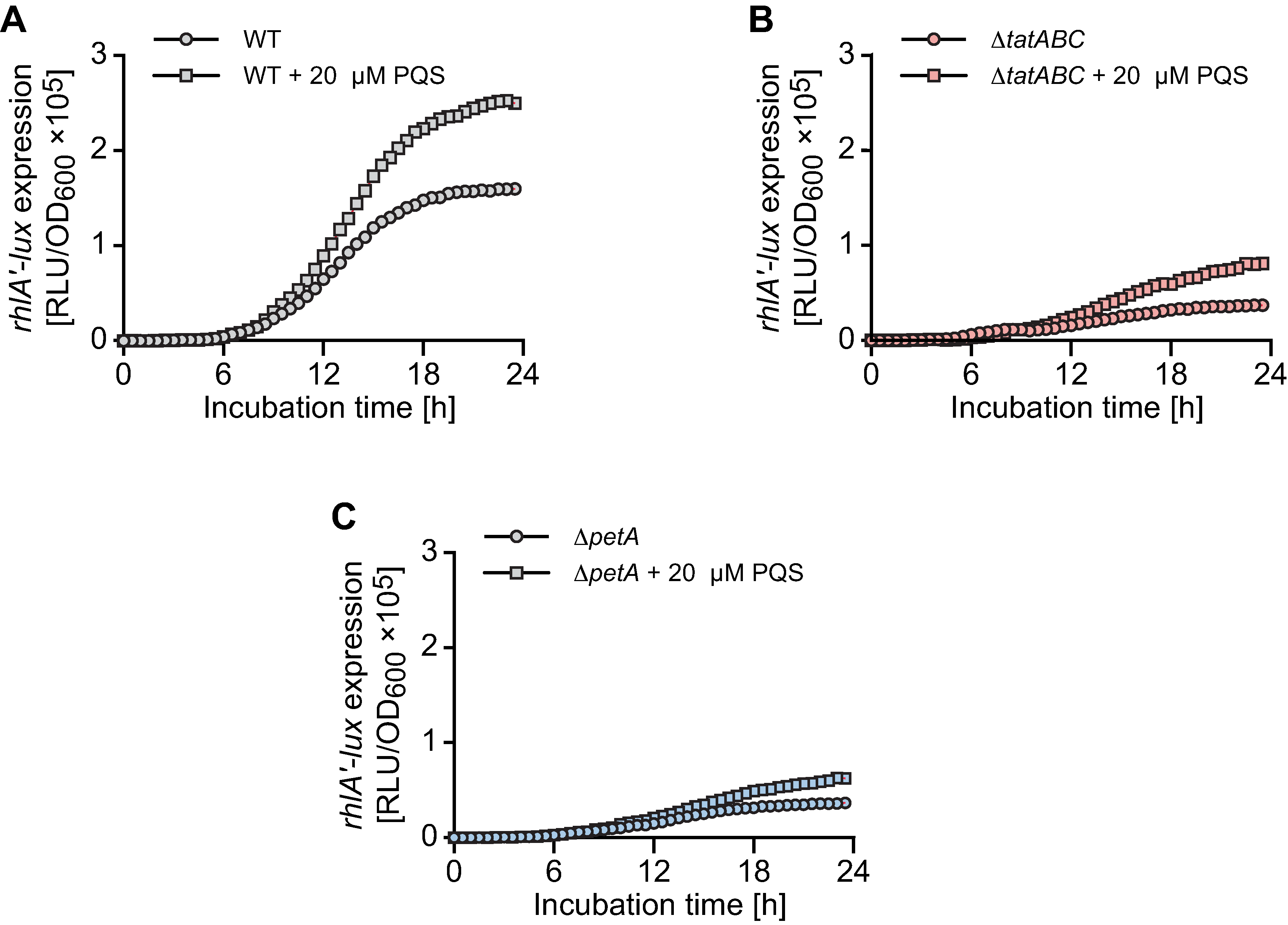

Supplement: S6 Fig — Bioluminescence from a chromosomal rhlA’-lux fusion as a function of growth (RLU/OD) over time when introduced into (A) the PA14 wild type, (B) ΔtatABC and (C) ΔpetA mutants in the absence or presence of exogenous PQS (20 μM). (TIF) [file ppat.1009425.s006.tif]

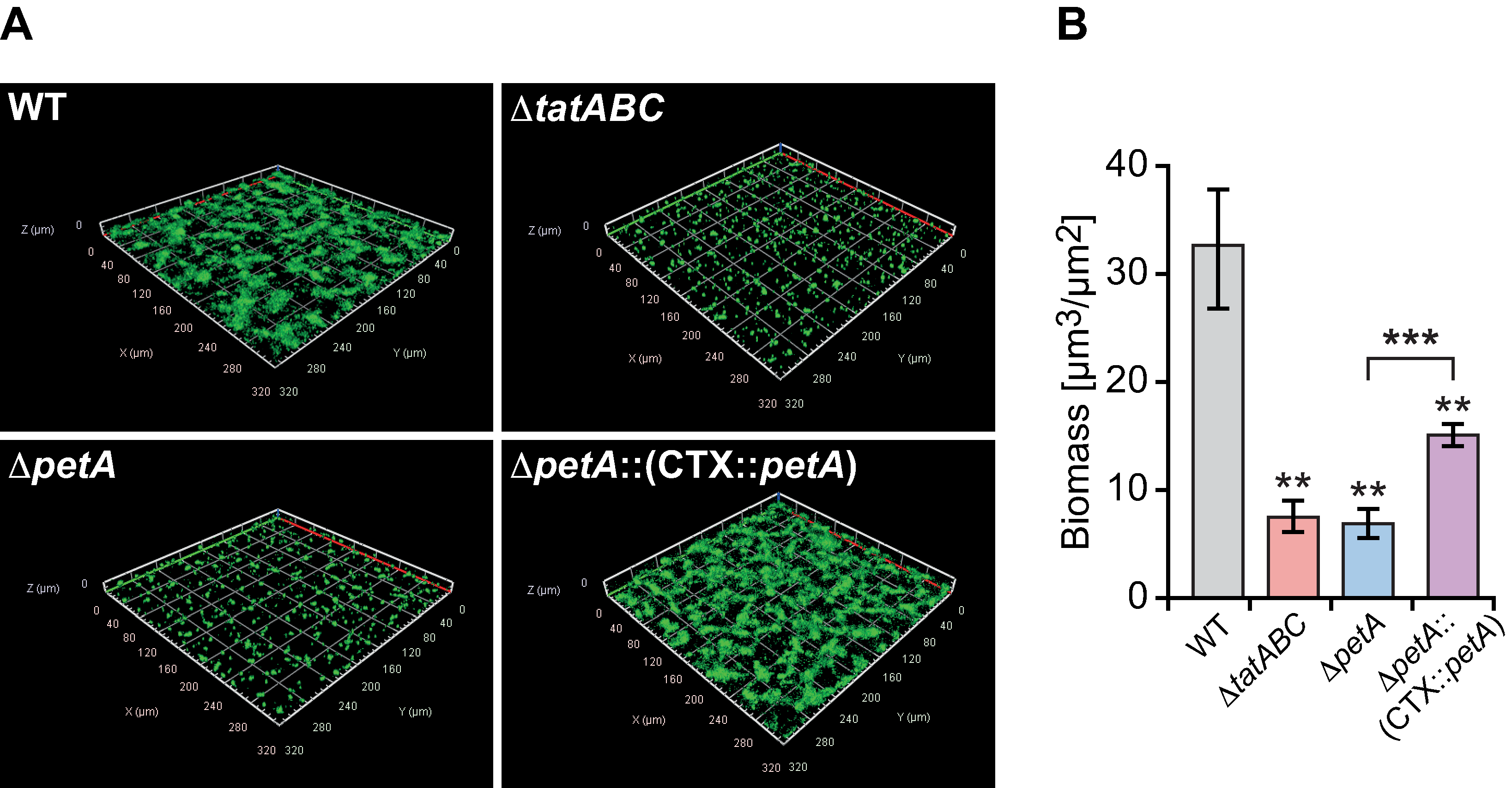

Supplement: S7 Fig — Biofilms of wild type PA14, the ΔtatABC and ΔpetA mutants and the genetically complemented PA14 mutant (ΔpetA::(CTX::petA)) were grown cultured statically and stained for eDNA with YOYO-1. (A) confocal fluorescence microscopy images and (B) eDNA quantification. Experiments were repeated in triplicate at least twice. ***p < 0.001, **p < 0.01. (TIF) [file ppat.1009425.s007.tif]

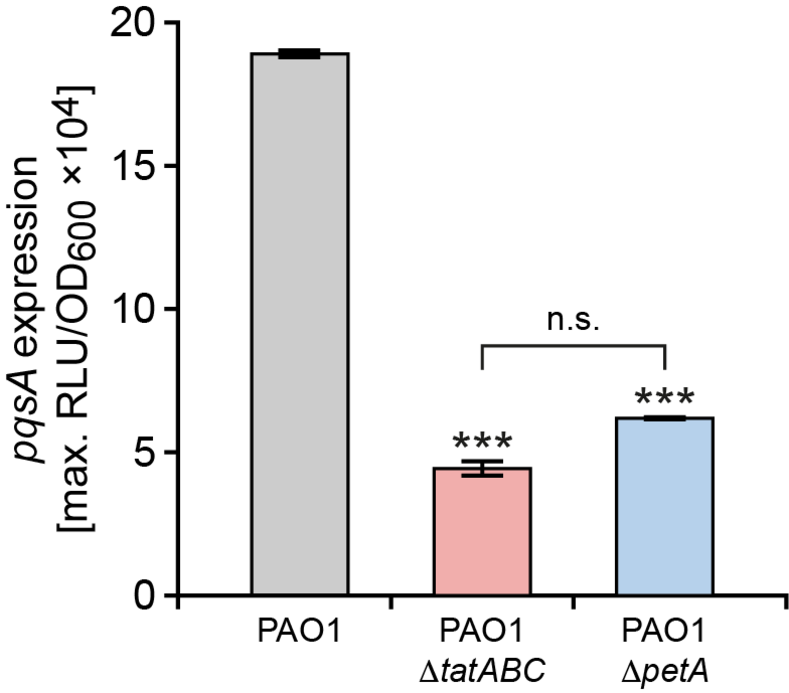

Supplement: S8 Fig — Experiments were repeated in triplicate at least twice. ***p < 0.001, **p < 0.01, and *p < 0.05; n.s. not significant. (TIF) [file ppat.1009425.s008.tif]

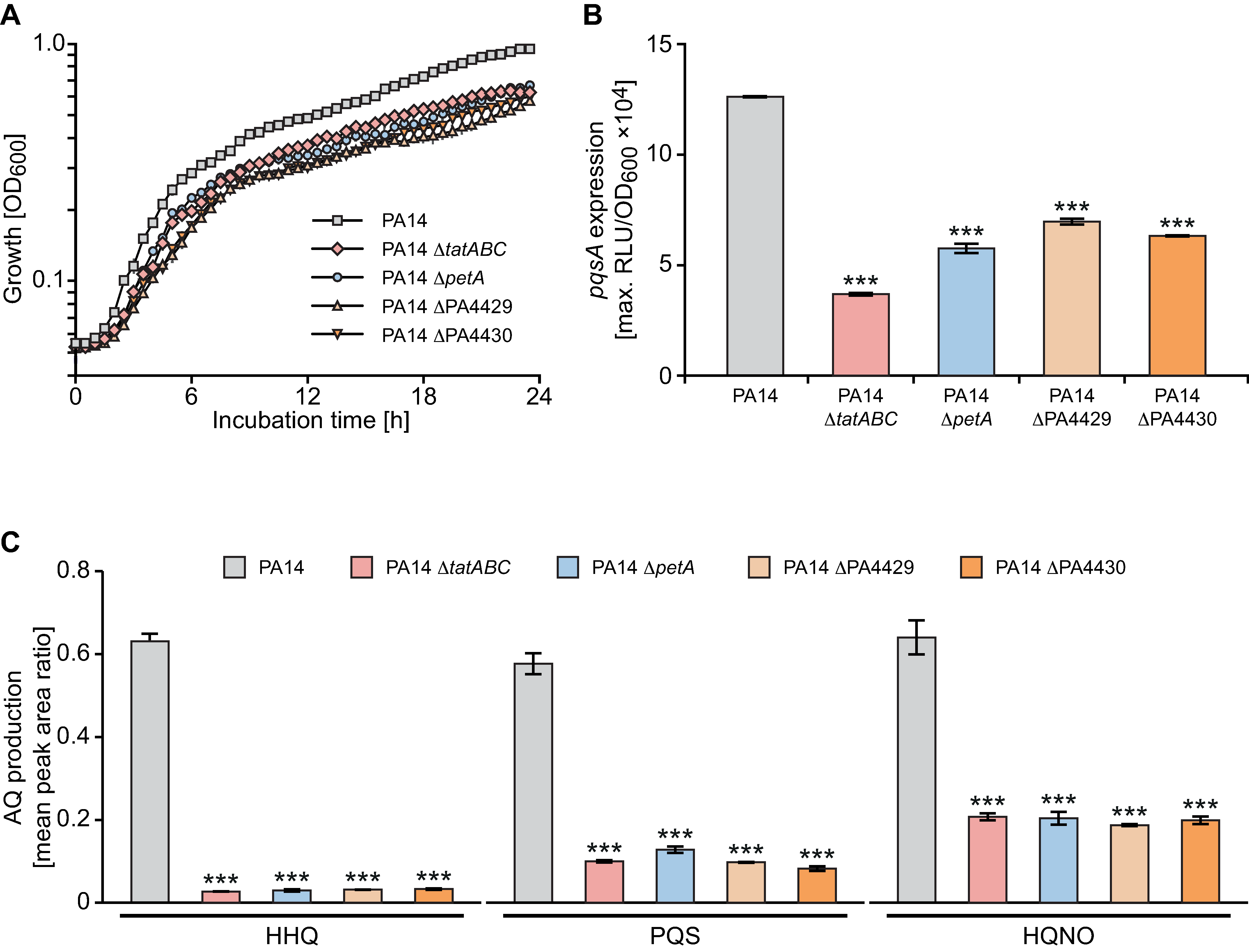

Supplement: S9 Fig — ***p < 0.001, **p < 0.01, and *p < 0.05; n.s. not significant. (TIF) [file ppat.1009425.s009.tif]

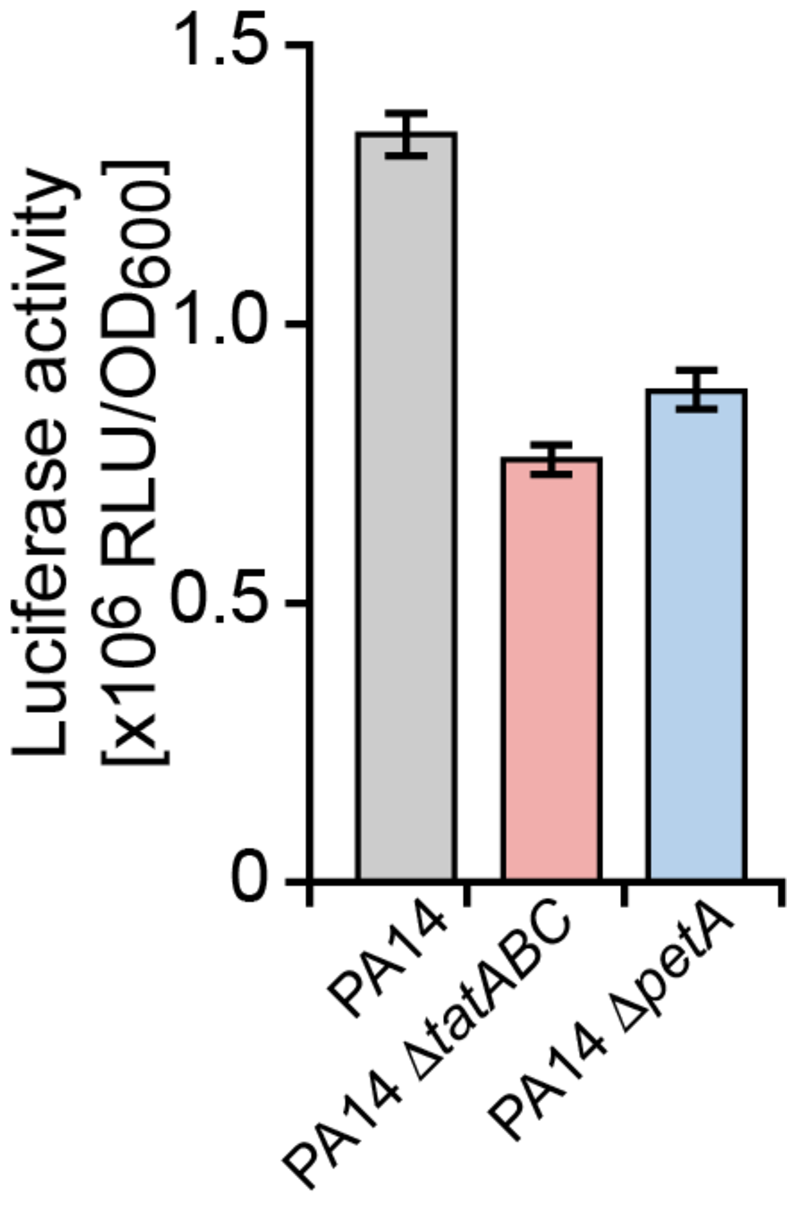

Supplement: S10 Fig — Experiments were repeated in triplicate at least twice. (TIF) [file ppat.1009425.s010.tif]
