# Supplementary material for: Disruption of the Pseudomonas aeruginosa Tat system perturbs PQS-dependent quorum sensing and biofilm maturation through lack of the Rieske cytochrome bc1 sub-unit
Source: PLoS Pathog. 2021 Aug 30;17(8):e1009425. doi: 10.1371/journal.ppat.1009425 (PMC8432897; doi:10.1371/journal.ppat.1009425)
Supplement: S1 Table — (DOCX) [file ppat.1009425.s011.docx]

**Table S1.** Strains and plasmids used in this study.

| **Strains/Plasmids** | **Relevant Characteristics** | **Source/Reference** |
| --- | --- | --- |
| ***E. coli*** |  |  |
| S17-1λ*pir* | Conjugative strain for suicide plasmids | [1] |
| ***P. aeruginosa*** |  |  |
| PAO1 | Wild-type *P. aeruginosa* strain | Copenhagen collection |
| PAO1 CTX::*pqsA'-lux* | PAO1 with a chromosomal miniCTX::*pqsA'-luxCDABE* fusion | [2] |
| PAO1 CTX::*rhlA'-lux* | PAO1 with a chromosomal miniCTX::*rhlA'-luxCDABE* fusion | This study |
| PAO1 CTX::*phzA1'-lux* | PAO1 with a chromosomal miniCTX::*phzA1'-luxCDABE* fusion integrated into the chromosomal CTX attachment site | [3] |
| PAO1 CTX::*phzA2'-lux* | PAO1 with a chromosomal miniCTX::*phzA2'-luxCDABE* fusion | [3] |
| PAO1 CTX::*pqsR'-lux* | PAO1 with a chromosomal miniCTX::*pqsR'-luxCDABE* fusion | This study |
| PAO1 CTX::*tac-lux* | PAO1 with a chromosomal miniCTX::*tac'-luxCDABE* fusion | This study |
| PAO1 Δ*pqsA* | *pqsA* in-frame deletion mutant; AQ-negative | [2] |
| PAO1 Δ*pqsA* CTX::*pqsA'-lux* | PAO1 Δ*pqsA* with a chromosomal miniCTX::*pqsA'-luxCDABE* fusion | [2] |
| PAO1 Δ*pqsA* CTX::*rhlA'-lux* | PAO1 Δ*pqsA* with a chromosomal miniCTX::*rhlA'-luxCDABE* fusion | This study |
| PAO1 *tatA* | *tatA* Himar 1 *mariner* transposon insertion mutant | This study |
| PAO1 Δ*tatABC* | *tatABC* in-frame deletion mutant | This study |
| PAO1 Δ*petA* | *petA* in-frame deletion mutant | This study |
| PAO1 *tatA* Δ*pqsA* | PAO1 *tatA* with a *pqsA* in-frame deletion | This study |
| PAO1 *tatA* CTX::*pqsA'-lux* | PAO1 *tatA* with a chromosomal miniCTX::*pqsA'-luxCDABE* fusion | This study |
| PAO1 *tatA* CTX::*rhlA'-lux* | PAO1 *tatA* with a chromosomal miniCTX::*rhlA'-luxCDABE* fusion | This study |
| PAO1 *tatA* CTX::*phzA1'-lux* | PAO1 *tatA* with a chromosomal miniCTX::*phzA1'-luxCDABE* fusion | This study |
| PAO1 *tatA* CTX::*phzA2'-lux* | PAO1 *tatA* with a chromosomal miniCTX::*phzA2'-luxCDABE* fusion | This study |
| PAO1 *tatA* Δ*pqsA* CTX::*pqsA'-lux* | PAO1 *tatA* Δ*pqsA* with a chromosomal miniCTX::*pqsA'-luxCDABE* fusion | This study |
| PA14 | Wild-type *P. aeruginosa* strain | This study |
| PA14 Δ*petA* | *petA* in-frame deletion mutant | This study |
| PA14 Δ*petA*::(CTX1::*petA*) | *petA* with a chromosomal miniCTX1*::petA* insertion, Tc^R^ | This study |
| PA14 Δ*cytB* | *cytB* in-frame deletion mutant | This study |
| PA14 Δ*cytC*_1_ | *cytC_1_* in-frame deletion mutant | This study |
|  |  |  |
| **Plasmids** |  |  |
| pBBR1MCS-5 | Broad host range vector, Gm^R^ | [4] |
| pBBR1MCS-5::*pqsABCD* | pBBR1MCS-5 carrying the *pqsABCD* operon | [5] |
| pBT20 | *Himar I mariner* mini-transposon delivery vector | [6] |
| pKNG101 | Suicide vector, Sm^R^, *oriR6K, oriTRK2, mobRK2, sacBR*^+^ | [7] |
| pKNGΔ*cytB* | Suicide plasmid for *cytB* deletion, Sm^R^ | This study |
| pKNGΔ*cytC_1_* | Suicide plasmid for *cytC_1_* deletion, Sm^R^ | This study |
| pKNGΔ*petA* | Suicide plasmid for *petA* deletion, Sm | This study |
| pME3087 | ColE1 suicide vector for allelic replacements, Tc^R^ | [8] |
| pME3087::*tatABC* | Suicide plasmid for *tatABC* deletion, Tc^R^ | This study |
| pME6032::*pqsR6H* | pME6032 carrying a functional, hexahistidine C-terminally tagged *pqsR gene* | [9] |
| pmini-CTX1 | mini-CTX delivery vector for integration of constructs at the *attB* site of *P. aeruginosa* chromosome; Tc^R^ | [10] |
| Pmini-CTX1-*petA* | *petA* under the control of its own promoter in mini-CTX1 | This study |
| pminiCTX-*lux* | Promoter probe vector containing *luxCDABE*, Tc^R^ | [11] |
| pminiCTX::*phzA1*’*-lux* | *phzA1* promoter region fused to *luxCDABE* in pminiCTX-*lux* | [3] |
| pminiCTX::*phzA2*’*-lux* | *phzA2* promoter region fused to *luxCDABE* in pminiCTX-*lux* | [3] |
| pminiCTX::*pqsA'-lux* | *pqsA* promoter region fused to *luxCDABE* in pminiCTX-*lux* | [2] |
| pminiCTX::*pqsR’-lux* | *pqsR* promoter region fused to *luxCDABE* in pminiCTX-*lux* | This study |
| pminiCTX::*rhlA'-lux* | *rhlA* promoter region fused to *luxCDABE* in pminiCTX-*lux* | This study |
| pminiCTX::*tac-lux* | *tac* promoter fused to *luxCDABE* in pminiCTX-*lux* | This study |
| pRK2013 | Conjugative helper plasmid, ColE1 origin, Tra^+^, mob^+^, Km^R^ | [12] |
| pTatA | pUCP22 carrying *tatA*; Ap^R^ | This study |
| pUCP22 | *E.coli -Pseudomonas* shuttle vector | [13] |
| pUCP*pqsE* | pUCP18 containing *pqsE*; Ap^R^ | [14] |

**References**

1. Simon R, Priefer U, Puhler A. A broad host range mobilization system for *in vivo* genetic engineering: transposon mutagenesis in Gram-negative bacteria. Nature Biotechnology. 1983; 1:784-91.

2. Diggle SP, Matthijs S, Wright VJ, Fletcher MP, Chhabra SR, Lamont IL, Kong X, Hider RC, Cornelis P, Cámara M, Williams P. The *Pseudomonas aeruginosa* 4-quinolone signal molecules HHQ and PQS play multifunctional roles in quorum sensing and iron entrapment. Chem Biol. 2007; 14:87-96.

3. Higgins S, Heeb S, Rampioni G, Fletcher MP, Williams P, Cámara M. Differential Regulation of the Phenazine Biosynthetic Operons by Quorum Sensing in *Pseudomonas aeruginosa* PAO1-N. Front Cell Infect Microbiol. 2018; 8:252.

4. Kovach ME, Elzer PH, Hill DS, Robertson GT, Farris MA, Roop RM 2nd, Peterson KM. Four new derivatives of the broad-host-range cloning vector pBBR1MCS, carrying different antibiotic-resistance cassettes. Gene. 1995; 166:175–176

5. Niewerth H, Bergander K, Chhabra SR, Williams P, Fetzner S. Synthesis and biotransformation of 2-alkyl-4(1H)-quinolones by recombinant *Pseudomonas putida* KT2440. Appl Microbiol Biotechnol. 2011; 91:1399-408.

6. Kulasekara HD, Ventre I, Kulasekara BR, Lazdunski A, Filloux A, Lory S. A novel two-component system controls the expression of *Pseudomonas aeruginosa* fimbrial cup genes. Mol Microbiol. 2005; 55:368-80.

7. Kaniga K., Delor I., Cornelis G. R. A wide-host-range suicide vector for improving reverse genetics in gram-negative bacteria: inactivation of the blaA gene of Yersinia enterocolitica. Gene*.* 1991; 109:137-41.

8. Schnider-Keel U, Lejbølle KB, Baehler E, Haas D, Keel C. The sigma factor AlgU (AlgT) controls exopolysaccharide production and tolerance towards desiccation and osmotic stress in the biocontrol agent *Pseudomonas fluorescens* CHA0. Appl Environ Microbiol. 2001; 67:5683-93.

9. Ilangovan A, Fletcher M, Rampioni G, Pustelny C, Rumbaugh K, Heeb S, Cámara M, Truman A, Chhabra SR, Emsley J, Williams P. Structural basis for native agonist and synthetic inhibitor recognition by the *Pseudomonas aeruginosa* quorum sensing regulator PqsR (MvfR). PLoS Pathog. 2013; 9:e1003508.

10. Hoang T. T., Kutchma A. J., Becher A., Schweizer H. P. Integration-proficient plasmids for Pseudomonas aeruginosa: site-specific integration and use for engineering of reporter and expression strains. Plasmid. 2000; 43:59-72.

11. Becher A, Schweizer HP. Integration proficient *Pseudomonas aeruginosa* vectors for isolation of single copy chromosomal *lacZ* and *lux* gene fusions. Biotechniques. 2000; 29:948-50.

12. Figurski D. H., Helinski D. R. Replication of an origin-containing derivative of plasmid RK2 dependent on a plasmid function provided in trans. Proc Natl Acad Sci U S A. 1979; 76:1648-52.

13. West SE, Schweizer HP, Dall C, Sample AK, Runyen-Janecky LJ. Construction of improved *Escherichia*-*Pseudomonas* shuttle vectors derived from pUC18/19 and sequence of the region required for their replication in *Pseudomonas aeruginosa*. Gene. 1994; 148:81-6.

14. Rampioni G, Pustelny C, Fletcher MP, Wright VJ, Bruce M, Rumbaugh KP, Heeb S, Cámara M, Williams P. Transcriptomic analysis reveals a global alkyl-quinolone-independent regulatory role for PqsE in facilitating the environmental adaptation of *Pseudomonas aeruginosa* to plant and animal hosts. Environ Microbiol. 2010; 12:1659-73.
