# Supplementary material for: Disruption of the Pseudomonas aeruginosa Tat system perturbs PQS-dependent quorum sensing and biofilm maturation through lack of the Rieske cytochrome bc1 sub-unit
Source: PLoS Pathog. 2021 Aug 30;17(8):e1009425. doi: 10.1371/journal.ppat.1009425 (PMC8432897; doi:10.1371/journal.ppat.1009425)
Supplement: S2 Table — (DOCX) [file ppat.1009425.s012.docx]

**Table S2.** Oligonucleotide primers used in this study

| **Name** | **Sequence** (5’-3’, restriction sites underlined) |
| --- | --- |
| TnM1 | GTGAGCGGATAACAATTTCACACAG |
| TnM2 | ACAGGAAACAGGACTCTAGAGG |
| TnMseq | CACCCAGCTTTCTTGTACAC |
| F*tatA* | GGAATTCCCCTGAACCTACACATTGCCA |
| R*tatA* | GGGGTACCCCATTCCGAACATCGATGGCTA |
| Tat3D-UF | TATGAATTCAATCTATTGGTCGCGTTC |
| Tat3D-UR | TATGGATCCAAAAATGCCCATGTCGTA |
| Tat3D-DF | TATGGATCCACCCGCCAGTGAACCTGC |
| Tat3D-DR | TATTCTAGACGCGCAGCTTGTCGACCT |
| S.PA4431UF | CAGGTCGACGGATCCCCGGGGGTACTGGCAAGATTTCCAT |
| S.PA4431DR | TATGCATCCGCGGGCCCGGGGGAGATCAGGAAGTCACCGC |
| S.PA4431DF | GACTGAATGTGATCGGCGTGGACCAGGAG |
| S.PA4431UR | CGCCGATCACATTCAGTCGTCTCCCATCA |
| S-4431CTXFor | TCCCCCGGGCTGCAGGAATTCTTCGACGCTTGCTGAAAA |
| S-4431CTXRev | GATAAGCTTGATATCGAATTCTCAGGCTTTCTCCTGGTC |
| S.PA4430UF | CAGGTCGACGGATCCCCGGGCCGTTCGTAGGGTCATGGTT |
| S.PA4430DR | TATGCATCCGCGGGCCCGGGACCACCTGTACCTGCTTGCA |
| S.PA4430DF | GAGAAAGCCTGATGAAAAAGCAATTCGCT |
| S.PA4430UR | TTGCTTTTTCATCAGGCTTTCTCCTGGTC |
| S.PA4429UF | CAGGTCGACGGATCCCCGGGATCGCGTTCCATCCGTACTA |
| S.PA4429DR | TATGCATCCGCGGGCCCGGGTCGCTGAGGAAATATGCCTT |
| S.PA4429DF | TGGCTGATGTAACCCGCACGTTGGTCTTC |
| S.PA4429UR | TGCGGGTTACATCAGCCAGTCACCCTTTC |
| *rhlA* CTX UF | TATAAGCTTTGCCAAAAGCCTGAC |
| *rhlA* CTX DR | TATGGATCCTTGCAAACCGATACC |
| *pqsR* CTX UF | TCCAGCGAATTCGATACGCAACCGCCG |
| *pqsR* CTX DR | GATGACCTGCAGGAACATGTTCACGTG |
| *tac* CTX UF | AAACTCCTCGAGCATCAAATGAAACTG |
| *tac* CTX DR | GAGCTCGAATTCTGTTTCCTGTGTGAA |
