# Supplementary material for: Disruption of the Pseudomonas aeruginosa Tat system perturbs PQS-dependent quorum sensing and biofilm maturation through lack of the Rieske cytochrome bc1 sub-unit
Source: PLoS Pathog. 2021 Aug 30;17(8):e1009425. doi: 10.1371/journal.ppat.1009425 (PMC8432897; doi:10.1371/journal.ppat.1009425)
Supplement: S3 Table — (DOCX) [file ppat.1009425.s013.docx]

**Table S3**. *P. aeruginosa* PA14 Tat substrate mutants used in this study

| **Mutant Number** | **PA Number/Name** | **Function** |
| --- | --- | --- |
| 1 | PA0144 | Nucleoside 2‑deoxyribosyltransferase |
| 2 | PA0365 | Hypothetical protein |
| 3 | PA0735 | Hypothetical protein |
| 4 | PA0844 | Hemolytic phospholipase C (PlcH) |
| 5 | PA1174 | Nitrate reductase catalytic subunit (NapA) |
| 6 | PA1601 | Aldehyde dehydrogenase |
| 7 | PA1880 | Oxidoreductase |
| 8 | PA2065 | Copper resistance protein (CopA) |
| 9 | PA2124 | Dehydrogenase |
| 10 | PA2264 | Hypothetical protein |
| 11 | PA2328 | Hypothetical protein |
| 12 | PA2378 | Aldehyde dehydrogenase |
| 13 | PA2389 | Hypothetical protein (PvdR) |
| 14 | PA2392 | Tyrosinase (PvdP) |
| 15 | PA2394 | Aminotransferase (PvdN) |
| 16 | PA2531 | Aminotransferase |
| 17 | PA2635 | Hypothetical protein |
| 18 | PA2699 | Hydrolase |
| 19 | PA3222 | Permease |
| 20 | PA3319 | Non-hemolytic phospholipase C (PlcN) |
| 21 | PA3392 | Nitrous-oxide reductase (NosZ) |
| 22 | PA3713 | Spermidine dehydrogenase (SpdH) |
| 23 | PA3768 | Metallo-oxidoreductase |
| 24 | PA3910 | Phosphodiesterase/alkaline phosphatase (EddA) |
| 25 | PA4140 | Cholesterol oxidase (ChoA) |
| 26 | PA4159 | Iron-enterobactin transporter periplasmic binding protein (FepB) |
| 27 | PA4431 | Cytochrome *bc*_1_ Rieske subunit (PetA) |
| 28 | PA4621 | Oxidoreductase |
| 29 | PA4692 | Sulphite oxidase subunit (YedY) |
| 30 | PA4812 | Formate dehydrogenase-O, major subunit (FdnG) |
| 31 | PA4858 | Hypothetical protein |
| 32 | PA5327 | Oxidoreductase (SphC) |
| 33 | PA5538 | N-acetylmuramoyl-L-alanine amidase AmiC* |
| 34 | PA14_48450 | Peptidyl-arginine deiminase (Agu2A’)** |

Annotations are based on predictions from the website www.pseudomonas.com or are inferred from sequence homology. Functional predictions by Gimenez *et al* [1].

*Annotated as AmiA on *Pseudomonas* PA14 genome but sequence is closer to that of AmiC.

**No conserved gene found in PAO1, so retains the PA14 annotation.

**Reference**

[1]. Gimenez MR, Chandra G, Van Overvelt P, Voulhoux R, Bleves S, Ize B. Genome wide identification and experimental validation of *Pseudomonas aeruginosa* Tat substrates. Sci Rep. 2018; 8:11950. doi: 10.1038/s41598-018-30393-x.
