# Supplementary material for: Disruption of the Pseudomonas aeruginosa Tat system perturbs PQS-dependent quorum sensing and biofilm maturation through lack of the Rieske cytochrome bc1 sub-unit
Source: PLoS Pathog. 2021 Aug 30;17(8):e1009425. doi: 10.1371/journal.ppat.1009425 (PMC8432897; doi:10.1371/journal.ppat.1009425)
Supplement: Attachment — Submitted filename: Responses to REviewers PLOS Pathogens 28072021.docx [file ppat.1009425.s014.docx]

**PLOS Pathogens PPATHOGENS-D-21-00412 – Response to Reviewers**

We thank the reviewers for their comments and help to improve our manuscript. We have extensively revised the text (see red track changes) throughout to improve the narrative and reduce repetition. We have responded to each point raised and included additional experimental data as follows:

**Reviewer #1: 
*The link between PetA, Tat and PQS is clearly made, however at present there is no molecular explanation for the observed phenotypes, leaving the reader unclear about how PetA affects PQS signalling. The authors need to come up with a model for the role of PetA (and the bc1 complex) to account for their findings*.**

We have now added additional data (**Figs S8, S9** and **S10)** and a working hypothesis for the links between Tat, PetA, cytochrome bc1 and PQS signalling e.g. see Discussion lines 574-593 and **Fig. S9** and as follows:

‘To uncover a direct link between Tat and PQS signalling, we screened a bank of Tat substrate mutants for reduced *pqsA* expression and identified PetA as primarily responsible for the PQS signalling defect. As PetA is the one of the three protein sub-units that constitute the cytochrome *bc_1_* complex, we also showed that a similar PQS signalling defect was apparent in *cytB* or *cytC_1_* mutants. Since growth of both the *tat* and cytochrome bc*_1_* subunit mutants was reduced compared with the wild type (**Fig. S9**), our working hypothesis is that shutting down secondary metabolism and virulence factor production would aid conservation of energy for primary metabolism and growth. This shutdown could be efficiently achieved by preventing PQS autoinduction since it co-ordinates the expression of multiple genes involved secondary metabolite production [17] including the biosynthesis of over 50 different AQs [54] that are heavily-dependent on tryptophan/anthranilate and fatty acid metabolic pathways. How PQS ‘shutdown’ is achieved at the molecular level in the *tat* mutants is not yet apparent but given that *tat ΔpqsA* double mutants do not respond to exogenous PQS, it may involve uptake into the cell and/or access to the response regulator PqsR(MvfR). At present there is little information on the mechanism by which PQS gains intracellular access although PQS is known to induce MV formation via a direct interaction with LPS and to be trafficked within MVs [33,34]. This is consistent with the reduction in MV production that we observed in the *tat* and *pqsA* mutants.

In addition, we previously presented an explanation for the lack of eDNA in *P. aeruginosa* biofilms based on the loss of autolysis mediated via HQNO poisoning of cytochrome bc1 given that the same AQ biosynthetic pathway generates both PQS and HQNO (See revised text, Discussion lines 594-628).

**Reviewer #2:** 

***The writing could use a bit more “storytelling” as there were times that I wasn’t sure where they were going****.*

We have revised and re-edited the paper to improve the narrative flow and reduce repetition.

**Reviewer #3**:

***No major summary points***

**Part II – Major Issues: Key Experiments Required for Acceptance**
Please use this section to detail the key new experiments or modifications of existing experiments that should be absolutely required to validate study conclusions. Generally, there should be no more than 3 such required experiments

**Reviewer #1**:

***Is this phenotype unique to the loss of PetA? Or is it phenocopied by deletion of genes encoding the other components of the bc1 complex? It would be important to test this.***

We have constructed additional strains with deletions in cytochrome b and c_1_ subunits and added new data (**Fig. S9**) to demonstrate that they exhibit the same phenotype as *tat* and *petA* mutants with respect to PQS signalling (specifically *pqsA* expression and AQ production)

***P. aeruginosa has other terminal oxidases that bypass the bc1 complex and take electrons directly from the quinone pool (e.g. CYO). If one of these is (over)expressed in the tat or petA mutant can the PQS defect be rescued?***

*P. aeruginosa* is well adapted for microaerobic respiration due to its branched aerobic respiratory chain terminated by five terminal oxidases; three are cytochrome c oxidases (cbb3-1, cbb3-2 and aa3), and two are quinol oxidases (Cyo and CIO). Hence while this would be interesting, we believe that it represents a considerable amount of extra work that is beyond the scope of the present paper.

***PQS and HHQ are chemically very similar to ubiquinone, and in particular to menaquinone. Is it possible that one or both of these molecules interact with the bc1 complex and are chemically modified in some way (for example by oxidation or reduction), and that this generates the active signalling molecule? The lack of exogenous complementation of the tat mutant by PQS is very telling in this regard and would be consistent with a role for the bc1 complex in generating the active form of PQS/HHQ. The other alternative for the lack of chemical complementation is that uptake of PQS is abolished (but given that overexpression of pqsABCD did not complement the tat mutant either this seems less likely).***

These are interesting points. However, PQS/HHQ are well established as the active signalling molecules as demonstrated by numerous extensive AQ structure activity relationship studies and by the structures of the crystal complexes of PqsR/AQs (see e.g. Ilangovan et al PLoS Pathogens). The only molecule generated via the same pathway as PQS/HHQ is the AQ N-oxide, HQNO (**new Fig. S1**) which is very well known as a cytochrome bc1 inhibitor as we discuss.

PQS signalling requires both export and import of PQS and HHQ molecules either directly or after packaging in MVs. Our data show that AQs do not accumulate intracellularly in the ΔpqsA Δtat double mutant harboring plasmid-borne *pqsABCD* genes in the absence of autoinduction (**original** **Fig. S4**) and that exogenous PQS added at inoculation does not fully restore *pqsA::lux* expression (**original Fig. 6**). These data are consistent with defective autoinduction rather than transport especially as AQ biosynthesis is not completely abolished in the *tat* mutant (**original Fig.** 6) but rather a failure of the PQS auto-induction circuitry to produce sufficient PQS/HHQ at the right time to efficiently activate *pqsABCDE*.

***Is the protonmotive force affected in the tat/petA mutant strains?***

We are not aware of any evidence to indicate that AQs require active transport. Tat transport requires pmf for protein export but mutation of *tat* is not known to affect pmf. In bacteria such as *Shewanella* (doi:10.1371/journal.pone.0062064) *tat* and *petA* mutants both show reduced aerobic growth compared with wild type. In the revised version, we now included growth curves for the *P. aeruginosa* *tat*, *petA cytB* and *cytC1* mutants compared with wild type (new **Fig. S9**) – slightly reduced growth is evident for each of the mutants.

**Reviewer #2**:

*No major comments*

**Reviewer #3:**

***First of all, it is unclear why most of the work is done in strain PAO1, while strain PA14 was chosen to make the 34 allelic replacement mutants. Isn’t there a PAO1 petA mutant? If not, why not construct it and then confirm that PetA is the Tat substrate that is responsible for perturbed PQS signaling, also in PAO1.***

This manuscript emerged from groups working independently - one on PQS/eDNA/Tat in PAO1 and the other on defining the repertoire of Tat substrates for which PA14 had been used. We came together to collaborate after the 34 PA14 mutants had already been constructed. In our original manuscript we showed that PAO1 and PA14 *tat* mutants behave very similarly. We have now constructed a PAO1 *petA* mutant and added new data (**Fig. S8**) to show that the PAO1 *petA* mutant behaves the same as the PA14 *petA* mutant with respect to *pqsA* expression.

***Secondly, a weakness of the study is that for the biofilm work only flow-cell grown biofilms are considered. This model has without any doubt its value, but at the same time it is also the only model in which we can observe ‘mushroom-shaped’ biofilms. I believe this study would be much stronger if its key findings could be confirmed in more in vivo-like conditions (e.g. biofilms formed in growth media resembling CF sputum or the wound environment).***

The reviewer has perhaps overlooked that we used both flow and static conditions as well as different media with the same results. The flow system allows comparison with many previous publications on *P. aeruginosa* biofilms and clearly shows consequences of the *tat* mutation. Therefore, although it would be interesting, we consider testing additional in vivo mimicking growth media to be beyond the scope of the present paper.

**Part III – Minor Issues: Editorial and Data Presentation Modifications**
Please use this section for editorial suggestions as well as relatively minor modifications of existing data that would enhance clarity.

**Reviewer #1**:

***Important details of the mariner Tn library are missing. There is no information on: 1) the size of the library (how many actual clones were produced), either before or after eliminating the double insertions; 2) how the double insertions were found; 3) how many clones passed selection and were identified as poor eDNA releasers; 4) the exact identity and frequency of all insertions found. How many of the 34 insertions were in pqs genes? This section is vaguely written and needs to be clarified. Can the authors comment on the reasons why no insertion in either tatC or petA were found? While this does not invalidate the authors’ subsequent investigation of the role of Tat/PetA, it may reflect incomplete coverage of the library, and this should be discussed.***

We did not attempt to construct a comprehensive defined Tn insertion library with the aim of complete chromosomal coverage but simply to discover Tn insertion mutants for reduced eDNA. We stated in our original manuscript that we screened 10,000 mutants, selected 84 with reduced levels of eDNA of which 34 mutants reproducibly showed the desired phenotype. These mutants were all in either *pqs* (*pqsC, pqsH* or *pqsR*) or *tat* genes (*tatA* and *tatB)*. Additional screening would likely have yielded further mutants but we chose to focus on the *tat* mutants. We do not have any other Tn mutants with eDNA defects.

***The title of the manuscript is not strictly accurate as the Rieske subunit is not lost in the tat mutant but mislocalized. Activity is lost.***

Since it is possible that the Rieske protein is either mislocalized or lost through degradation in the *tat* mutant, we have amended the title to ‘Disruption of the *Pseudomonas aeruginosa* Tat system perturbs PQS-dependent quorum sensing and biofilm maturation through lack of the Rieske cytochrome *bc_1_* sub-unit’

***Please add anthranilate to Fig 1 as the precursor for HHQ/PQS biosynthesis***

As this would over complicate **Fig. 1** and not account for the multiple other substrates involved or HQNO biosynthesis we have added an additional figure (**Fig. S1**) showing the biochemical pathway for AQ/AQ-N-oxide biosynthesis.

***Do the tat or petA mutants grow more slowly than the wild type strains? This would be useful information to provide as it might impinge on some of the findings.***

We have now included growth curves for the *tat, petA cyctB* and *cytC1* mutants (**Fig. S9A**) as explained above in response to reviewer #1’s first query about the mechanistic link between *petA* and PQS signalling.

***The small molecule inhibitor Tat data does not add anything and should be removed. It should be noted that Bageshwar et al (PLoS ONE 2016*** [***https://doi.org/10.1371/journal.pone.0149659***](https://doi.org/10.1371/journal.pone.0149659)***) concluded that although Bayer-11 7082 inhibited growth under Tat++ conditions it did not affect Tat transport in vitro.***

The work described by Bageshwar et al is on the *E. coli* Tat system. The authors indicate that Bayer-11 7082 inhibited *E. coli* growth. They did not test the compound on *P. aeruginosa* Tat in cells or in vitro and state they could not rule out that Bayer-11 7082 is a Tat inhibitor for *P. aeruginosa* but not for *E. coli*. Our data are consistent with that of Vasil et al 2012. The compound was not growth inhibitory at the concentrations used for *P. aeruginosa* nor does it inhibit the *pqsA* reporter luciferase itself (see revised manuscript **Fig. S2**). We have retained that data as it importantly confirms our observations with the *pqsA::lux* fusions.

***It is clear that PetA alone has major effect in biofilm development and associated events. However, of the other 30+ Tat substrates many have small but, presumably, cumulative contributions, so they authors should acknowledge this by clarifying that PetA is the major factor rather than the sole factor affecting biofilm formation****.*

In the revised version we have indicated that PetA is the ‘primary’ or ‘primarily’ Tat substrate responsible. See revised version Abstract, line 52 and line 628.

**The discussion is very long and to in places is repetitious of the results section. It should be shortened. The authors speculate on the role of PetA in leading to cell bursts and eDNA release – what is the evidence that ROS production is the means, by which PetA contributes to these events?**

We have revised and shortened the Discussion. HQNO disrupts flow of electrons through respiratory chain at bc1 complex resulting in donation of electrons to O_2_ and formation of ROS – *petA* mutants are insensitive to HQNO mediated autolysis. See the very extensive experimental work on this published by Hazan et al (cited as revised reference **57**)

**Reviewer #2**: Comments.

**1**. ***Line 225.* *A line should be added to explain the significance of a tatA mutant being more sensitive to tobramycin.***

Lines 235-239 now include the statement: Since eDNA binds positively charged antibiotics and as exogenously provided DNA increases aminoglycoside tolerance by integrating into *P. aeruginosa* biofilms, the increased sensitivity to tobramycin is likely to be a consequence of the reduction in eDNA within the biofilm extracellular matrix [46, 47].

**2.** ***Figure S3 should be a regular figure as it is important****.*

**Fig. S3** is now **Fig. 8** in the revised manuscript.

**3. Line 276. There should be a line of discussion for this result. Also – same issue at line 293.**

We have re-ordered these results sections. For lines 293-294: We have added as a discussion comment : ‘This reduced response to PQS could be due either to reduced uptake or the inability to respond to the exogenous QS signal molecule.’ This is then taken up and qualified in the revised Results lines 315-331. ‘These data suggested that in a *tat* mutant background, the lack of AQs was not a consequence of an AQ export defect but due to the inability to activate AQ biosynthesis. In the absence of an auto-inducible *pqs* system in a *tat* mutant background, the defect in AQ biosynthesis and hence *pqs* signalling appears to be more severe’.

**4**. ***The discussion has a lot of restating of results.***

We have revised and shortened the Discussion to reduce repetition

**5. *The meaning of the tatA mutant isn’t touched upon until line 481. It should be explained much earlier and reiterated in the section at line 481.***

We have introduced the Tat system at the first time of mention in the revised Results section lines 205-211:

Apart from the *pqs* mutants, two mutants were obtained with insertions in the *tatA* and *tatB* genes respectively (**Fig. 2**) that code for components of the twin-arginine translocation (Tat) system. Tat exports folded proteins out of the cytoplasm and across the cytoplasmic membrane in an ATP-independent manner [42]. It was originally named with respect to the presence of an Arg-Arg motif in the signal sequence of Tat-exported products (sometimes called Tat substrates) [42,43]. In *P. aeruginosa* the Tat translocase complex consists of three proteins (TatA,B and C) [43]. TatB and TatC form the receptor complex for Tat substrate precursors whereas TatA functions as the main facilitator for protein translocation across the membrane [42,43].

**Reviewer #3:**

***Finally, while reading the manuscript it felt like there was a lot of repetition in the Discussion and I would suggest to consider combining Results and Discussion.***

We have revised and shortened the Discussion to reduce repetition
